# Finite Element Modeling of Abdominal Near‐Infrared Spectroscopy for Infant Splanchnic Oximetry

**DOI:** 10.1002/cnm.70035

**Published:** 2025-04-15

**Authors:** Vishnu S. Emani, Caglar Ozturk, Manisha Singh, Carly Long, Summer Duffy, Danielle Gottlieb Sen, Ellen T. Roche, Wesley B. Baker

**Affiliations:** ^1^ School of Engineering and Applied Sciences Harvard University Cambridge Massachusetts USA; ^2^ Institute for Medical Engineering and Science, Massachusetts Institute of Technology Cambridge Massachusetts USA; ^3^ Department of Mechanical Engineering University of Southampton Southampton UK; ^4^ Department of Mechanical Engineering Massachusetts Institute of Technology Cambridge Massachusetts USA; ^5^ Division of Pediatric Cardiac Surgery Johns Hopkins University Baltimore Maryland USA; ^6^ Division of Neurology, Department of Pediatrics Children's Hospital of Philadelphia and University of Pennsylvania Philadelphia Pennsylvania USA

**Keywords:** diffusion processes, near‐infrared spectroscopy, necrotizing enterocolitis, splanchnic oximetry, tissue optics

## Abstract

Abdominal near‐infrared spectroscopy (NIRS) holds promise for early detection of necrotizing enterocolitis and other infant pathologies prior to irreversible injury, but the optimal NIRS sensor design is not well defined. In this study, we develop and demonstrate a computational method to evaluate NIRS sensor designs for infant splanchnic oximetry. We used a finite element (FE) approach to simulate near‐infrared light transport through a 3D model of the infant abdomen constructed from computed tomography (CT) images. The simulations enable the measurement of the contrast‐to‐noise ratio (CNR) for splanchnic oximetry, given a specific NIRS sensor design. A key design criterion is the sensor's source–detector distance (SDD). We calculated the CNR as a function of SDD for two sensor positions near the umbilicus. Contrast‐to‐noise was maximal at SDDs between 4 and 5 cm, and comparable between sensor positions. Sensitivity to intestinal tissue also exceeded sensitivity to superficial adipose tissue in the 4–5 cm range. FE modeling of abdominal NIRS signals provides a means for rapid and thorough evaluation of sensor designs for infant splanchnic oximetry. By informing optimal NIRS sensor design, the computational methods presented here can improve the reliability and applicability of infant splanchnic oximetry.

## Graphical abstract

In this study, we developed a computational method to evaluate near‐infrared spectroscopy sensor designs for infant splanchnic oximetry. We used a finite element model to simulate near‐infrared light transport through a 3D model of the infant abdomen, and calculated contrast‐to‐noise ratio (CNR) as a function of source–detector distance (SDD) to measure sensor performance. Our results suggested that SDDs between 4 and 5 cm optimize CNR and thus may be optimally suited for splanchnic oximetry.

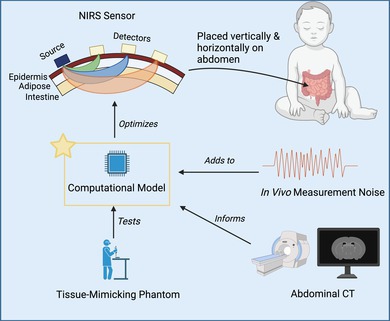

## 1 Introduction

Near‐infrared spectroscopy (NIRS) and related diffuse optical techniques have been used with great success over the last two decades to noninvasively probe regional oxygen delivery in human tissues. Although the majority of clinical NIRS development to date has focused on the brain [1, 2, 3, 4], breast [5, 6], and skeletal muscle [7, 8, 9], there is growing interest in abdominal NIRS. Pilot studies have shown the promise of abdominal NIRS for assessing placental insufficiency in pregnant mothers [10, 11, 12], adipose metabolism during weight loss [13], and splanchnic oximetry [14, 15, 16].

As such, there is considerable interest in abdominal NIRS for early prediction of necrotizing enterocolitis (NEC) in infants. NEC, marked by localized or widespread injury of intestinal tissue, is a major cause of morbidity and mortality in preterm infants and in infants with congenital heart defects [17, 18, 19, 20]. Unfortunately, the current diagnosis of NEC often occurs at irreversibly late stages in NEC pathogenesis [20, 21, 22, 23, 24]. An early diagnostic of NEC via NIRS thus holds promise to inform clinical management and improve outcomes. The use of abdominal NIRS is justified by the hypothesis that NEC may be linked with reduced splanchnic perfusion and intestinal ischemia, especially in the case of congenital heart disease [25, 26].

Preliminary research suggests that abdominal NIRS can detect intestinal ischemia via its estimate of diminished blood oxygen saturation in the splanchnic circulation. One such study found that abdominal NIRS measurements were significantly lower in preterm infants who developed NEC compared to healthy controls [27]. Other studies in piglet models have also demonstrated correlations between abdominal NIRS oximetry and intestinal ischemia and injury [16, 28]. NIRS is thus a promising diagnostic technology for NEC, but the optimal NIRS sensor design configurations, including position on the abdomen and source–detector distance (SDD), are not well defined for splanchnic oximetry.

In order to provide a means for rapid evaluation of different NIRS sensor designs and implementations, we developed a method to simulate abdominal NIRS signals from infants for a range of potential sensor configurations. The method uses a finite element (FE) algorithm [22, 29, 30] to computationally predict the NIRS fluence rate signal in the infant abdomen. The model was built from clinical imaging data from a normal infant abdomen to provide realistic anatomy, and in vivo measurement noise was overlayed on the model data. The method was used to evaluate different SDDs in sensors placed vertically and horizontally near the umbilicus. This computational method promises to inform the design and implementation of abdominal NIRS for clinical monitoring applications.

## 2 Methods

**FIGURE 1:**
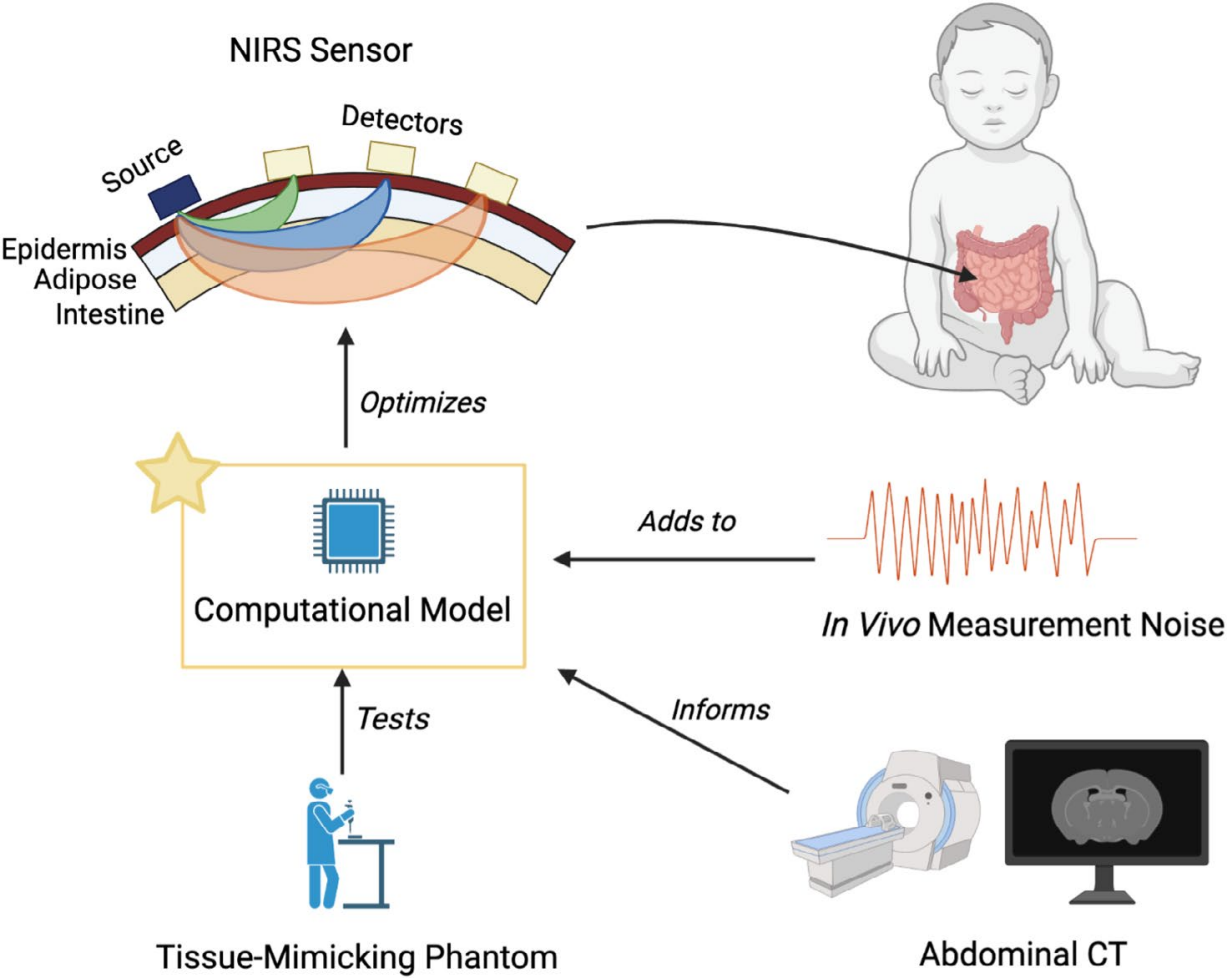
Schematic diagram of the study's workflow. The focus, indicated by the star, was developing a computational model of near‐infrared light transport through abdominal tissues (i.e., epidermis, adipose, intestine), which we used to identify optimal NIRS source–detector distances for intestinal monitoring at vertically and horizontally oriented sensor positions that avoid the rib cage and umbilicus structures (sensor positions shown in Figure 2). Note that measurements at longer source–detector distances sample deeper tissue volumes (roughly shown by the schematic “banana patterns” from source to detector), but have lower signal‐to‐noise. Optimization was based on contrast‐to‐noise ratio estimates derived from in vivo measurement noise added to the computational model.

A diagram of our methodological approach to simulate abdominal NIRS fluence rate signals for a given sensor design is shown in Figure 1. All study procedures were approved by the Johns Hopkins University Institutional Review Board (Protocol No. IRB00316916, approved April 13, 2022). Patient consent was waived by the Institutional Review Board due to the use of de‐identified imaging data for this study. Our computational model simulates NIRS fluence rates via FE solutions to the photon diffusion equation that describes light transport through tissue (Section 2.1). To support the model's validity and to enable the addition of in vivo noise for estimating contrast‐to‐noise ratio (CNR), we compared the FE solution in a rectangular homogeneous geometry to in vitro fluence rate measurements obtained in the same geometry (Section 2.2). Abdominal NIRS fluence rates were simulated using FE solutions to a heterogeneous geometry built from an abdominal computed tomography (CT) scan of an infant (Section 2.3). Specifically, the fluence rates at multiple point detectors arranged on a single line from a point source (or emitter) were simulated; the SDDs were between 1 and 10 cm. The “baseline” fluence rate as a function of SDD was obtained based on the assignment of physiologically reasonable optical properties (Section 2.4). Then, three corresponding sets of “perturbed” fluence rates were obtained by individually modifying the optical absorption of intestinal tissue, superficial adipose tissue, and epidermal tissue. To identify optimal SDDs for splanchnic oximetry, the NIRS sensitivity and CNR for the intestinal layer were computed from the baseline and perturbed fluence rates (Sections 2.5 and 2.6).

### 2.1 NIRS Signal Model

Near‐infrared light transport over long distances in highly scattering tissues is well approximated as a diffusive process [22]. Accordingly, we modeled the photon fluence rate ($\phi \left(\vec{r}\right)$) for continuous‐wave sources via the photon diffusion equation:

(1) $$-\nabla \cdot \left(D\left(\vec{r}\right)\nabla \phi \left(\vec{r}\right)\right)+\mu_{a}\left(\vec{r}\right)\phi \left(\vec{r}\right)=S\left(\vec{r}\right).$$

Here, $\vec{r}$ is the spatial position in tissue, $S\left(\vec{r}\right)$ is the source intensity (in W/cm^3^), $\phi \left(\vec{r}\right)$ is the photon fluence rate (in W/cm^2^), and $D\left(\vec{r}\right)=\frac{1}{3\left(\mu_{a}\left(\vec{r}\right)+\mu_{s}^{\prime }\left(\vec{r}\right)\right)}$ is the photon diffusion length (in cm), which depends on the tissue optical absorption coefficient $\mu_{a}\left(\vec{r}\right)$ (in cm^−1^) and reduced scattering coefficient $\mu_{s}^{\prime }\left(\vec{r}\right)$ (in cm^−1^).

We adopted the widely accepted and validated Robin boundary condition (also known as the partial flux boundary condition) at the air–tissue interface to solve the photon diffusion equation [22, 31], by which the gradient of fluence rate at the boundary is proportional to the fluence rate:

(2) $$\phi =z_{b}\hat{n}\cdot \nabla \phi ,$$

where $z_{b}=2l_{\mathrm{tr}}\left(1+R_{\mathrm{eff}}\right)/3\left(1-R_{\mathrm{eff}}\right)$, $R_{\mathrm{eff}}$ is the effective Fresnel reflectance at the boundary, $l_{\mathrm{tr}}$ is the transport mean‐free path, approximated as $1/\mu_{s}^{\prime }$, and the unit vector $\hat{n}$ points from inside the tissue to outside (the vector is perpendicular to the tissue surface).

We used a FE method to solve the photon diffusion equation with the Robin boundary condition [22, 31]. Specifically, we used the implementation of the method in the COMSOL Multiphysics 5.3 MUMPS numerical solver. The inputs to the numerical solver were (1) a mesh of tissue spatial positions, $\vec{r}$, with $\mu_{a}\left(\vec{r}\right)$ and $D\left(\vec{r}\right)$ specified, (2) an isotropic point source specified, that is, $S\left(\vec{r}\right)=S_{0}\delta \left(\vec{r}-{\vec{r}}_{s}\right)$, where $\delta \left(\vec{r}-{\vec{r}}_{s}\right)$ is the delta function centered at position ${\vec{r}}_{s}$ on the mesh, and (3) the specification of the Robin boundary condition at the boundaries of the mesh. The abdominal mesh, that is, input 1, is described in Section 2.3. For input 2, the source intensity was $S_{0}=1\;W$ and the ${\vec{r}}_{s}$ location was at a distance of $l_{\mathrm{tr}}$ directly underneath the source on the mesh boundary; an isotropic source at this location is an excellent approximation of the directional light from a source on the tissue surface [22]. For input 3, we used the $R_{\mathrm{eff}}=0.493$ for an air–tissue boundary [31] to specify the Robin boundary condition.

### 2.2 Optical Phantom Test

To test the FE method, we prepared simple phantom solutions with known optical properties, that is, five 1% Intralipid emulsion solutions were mixed with Higgins India ink of various concentrations. Intralipid and India ink are commonly used for biological optical phantoms because they present absorption and scattering properties in a physiological range [3]. We used India ink concentrations ranging from 0.0042% to 0.0252%, which, based on previous measurements performed with the same brand and concentrations of ink [19], have absorption coefficients ranging from 0.25 to 1 cm^−1^. Also, based on prior measurements [32], the reduced scattering coefficient of 1% intralipid emulsion is 10 cm^−1^.

Each intralipid emulsion was placed in a flat acrylic tube (1 in. × 1 in. × 3 ft) at 22°C, and the fluence rate emerging from the emulsion at 1.0 cm SDD was measured with a commercial NIRS device (BIOPAC Systems Inc., Goleta, CA, USA; note, the sensor only had one SDD at 1 cm, which unfortunately prevented testing at longer SDD). Specifically, the measurement was 3 min long (at an acquisition rate of 1 Hz), and each tube was measured three times. The mean and standard deviation of the fluence rate measurements across the 9 min of data for each tube were computed. All measurements were performed within less than an hour after the intralipid emulsions were made, such that the ink was in stable suspension, and no agitation of the emulsions was performed during the measurements.

These in vitro measurements were compared to the FE solutions of the fluence rate obtained with the COMSOL numerical solver for the equivalent rectangular geometry and optical properties (see Section 2.1). We normalized the in vitro and FE fluence rates to the numerical values at the lowest absorption for comparison. Note that the ratio of the measured fluence rate to FE fluence rate at the lowest absorption was the light coupling coefficient used for the CNR computations (see Section 2.6).

### 2.3 Abdomen Geometry

**FIGURE 2:**
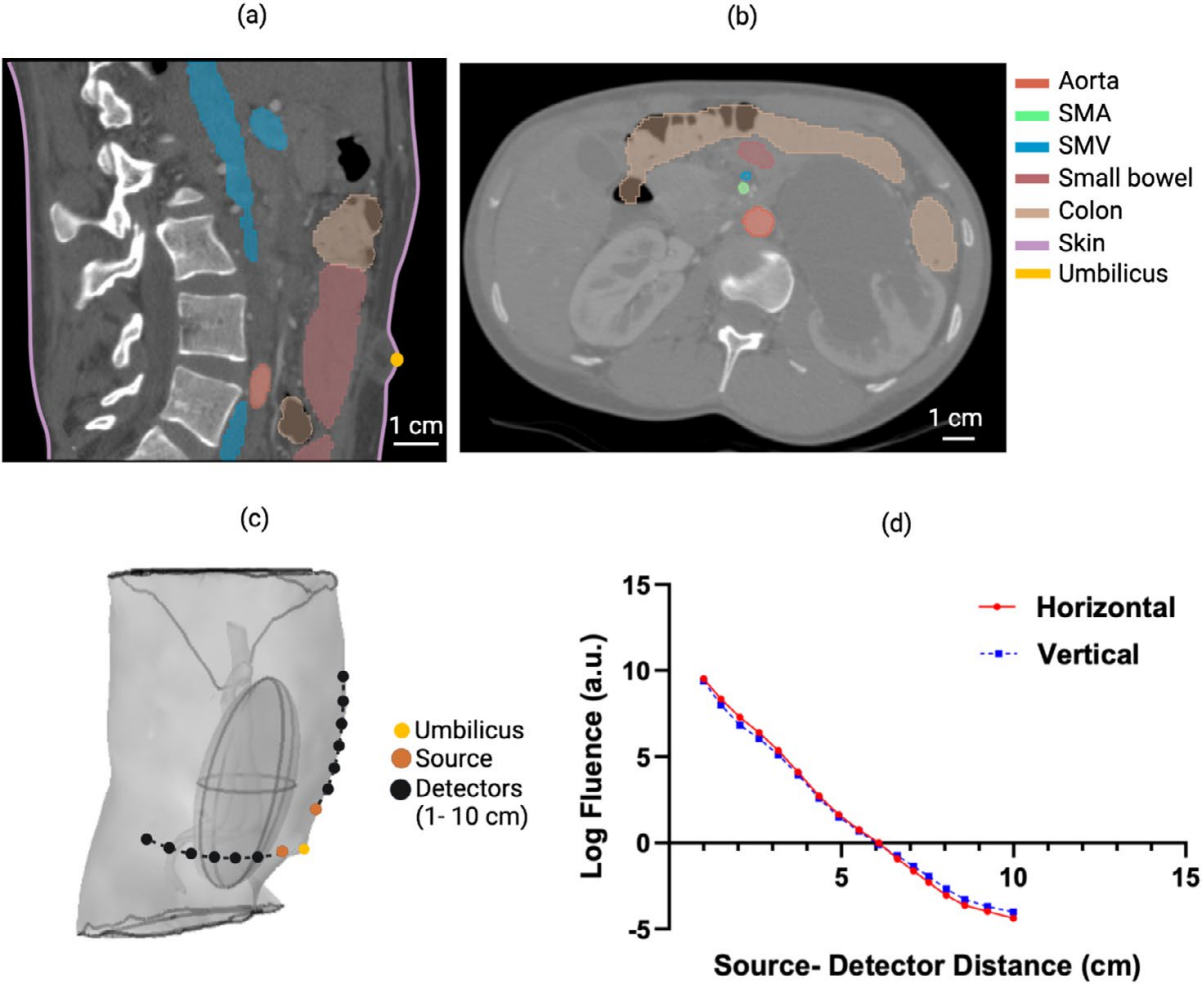
The process of building 3D abdominal models from a CT scan. (a) Sagittal and (b) axial CT cross sections of a 10‐day‐old healthy infant (SMA and SMV, Superior Mesenteric Artery and Vein); (c) the intestine was approximated as a homogeneous ellipsoid, and two sensor positions were simulated (vertical orientation with source 1 cm superior to umbilicus; horizontal orientation with source 1 cm lateral to umbilicus). (d) The natural logarithm of the simulated baseline photon fluence rate plotted against source–detector distance (SDD) for both sensor positions.

We reconstructed a 3D model of the abdomen by segmenting CT scans of a healthy infant (10 days old, 3.8 kg; Figure 2a). The segmentation included the Superior Mesenteric Artery (SMA), Superior Mesenteric Vein (SMV), adipose tissue, and the intestine (Figure 2b; the SMA and SMV were truncated from 3 cm superior to the intestine). To decrease computational complexity, we excluded the renal and hepatic veins; they will have minimal effect on the NIRS readings for the two sensor positions simulated due to their anatomical locations, which are distant from the source and detectors positioned near the umbilicus [20]. To model the microvasculature perfusing the intestine, we modeled the intestinal tissue as a homogeneous ellipsoid surrounding the intestine (thickness 2.6 cm, center of volume 3.4 cm beneath the skin, angle of major axis at 10° from superior–inferior axis; Figure 2c).

We then segmented a 0.008 cm‐thick epidermal layer directly beneath the skin surface in the CT image. The tissue between the intestinal ellipsoid structure and the epidermal layer was modeled as homogeneous adipose tissue (accordingly, abdominal muscle, adipose tissue, and other peripheral tissues are treated as a single homogeneous tissue layer). We applied standard tetrahedral meshing, resulting in approximately 900,000 domain mesh elements and 160,000 air–tissue boundary elements. NIRS measurements were modeled for two positions (Figure 2c): (a) the NIRS point source was placed 1 cm superior to the umbilicus, and 10 point detectors were placed along a vertical line above the source at SDDs between 1 and 10 cm, and (b) the NIRS point source was placed 1 cm lateral to the umbilicus, and 10 point detectors were placed along a horizontal line lateral to the source at SDDs between 1 and 10 cm. For the superior placement, the depths of the intestinal ellipsoid surface underneath the sensor ranged from 2.3 to 2.6 cm across all detectors, and from 2.2 to 2.5 cm for the lateral placement. These two sensor positions were chosen because they avoid the umbilicus and rib cage and thus mitigate potential measurement confounds from these anatomic structures.

We performed simulations for different adipose layer thicknesses of 2, 2.2, 2.4, 2.6, and 2.8 cm, where thickness was measured as the shortest distance from the skin boundary to the surface of the intestinal ellipsoid; the true thickness from the CT scan segmentation was 2.4 cm. These variations were obtained by compressing or stretching the abdominal cavity geometry in the anterior–posterior direction, while the dimensions for the intestines, SMA, and SMV remained constant. Based on the assigned tissue optical properties in this model (see Sections 2.4 and 2.5), the FE solutions for the photon fluence rate for each SDD and each sensor position were calculated, as described in Section 2.1. The photon fluence rates versus SDD for the “baseline” optical properties (Section 2.4) are shown for the adipose thickness of 2.4 cm at both sensor positions in Figure 2d.

### 2.4 Tissue Optical Properties

We assigned distinct optical absorption and reduced scattering coefficients at the 750 nm wavelength for the SMA, SMV, microvascular structures within the intestinal tissue (demarcated by the ellipsoid structure in Figure 2c), adipose tissue layer, and epidermal tissue layer (Table 1). We chose 750 nm for our simulations because it is a common wavelength used for NIRS, and its use in the accurate reconstructions of oxy‐ and deoxy‐hemoglobin concentrations has been demonstrated theoretically and experimentally [22, 23]. The computational model remains valid for other near‐infrared wavelengths. For the intestine, adipose, and epidermal layers, the assigned optical properties were based on prior measurements from the literature at the 750 nm wavelength (Table 1). For the SMA and SMV, the Beer–Lambert law was used to derive optical absorptions based on assumed blood oxygen saturation (SO_2_) levels and an assumed blood hemoglobin concentration ([Hb]) of 14.1 g/dL, that is,

(3) $$\mu_{a}=\left[\mathrm{Hb}\right]\;\left(\epsilon_{\mathrm{oxy}}\cdot SO_{2}+\epsilon_{\text{deoxy}}\cdot \left(1-SO_{2}\right)\right).$$

Here, $\epsilon_{\mathrm{oxy}}$ and $\epsilon_{\text{deoxy}}$ represent the extinction coefficients of oxy‐ and deoxy‐hemoglobin, respectively, at 750 nm [24], and SO_2_ was assumed to be 98% for the SMA and 75% for the SMV. The reduced scattering coefficient assigned for the SMA and SMV was based on literature measurements.

**TABLE 1:** Baseline optical properties.

Tissue	Absorption coefficient (cm^−1^)	Reduced scattering coefficient (cm^−1^)	Sources
Subcutaneous adipose	0.05	20	Lanka et al. [33] Ganesan et al. [13] Shimojo et al. [34] Fredriksson et al. [35]
Epidermis	1.2	38	Shimojo et al. [34] Fredriksson et al. [35]
SMA	2.5	25	Bosschaart et al. [36]
SMV	4.0	25	Bosschaart et al. [36]
Intestine	0.21	5	Sandell et al. [37] Friebel et al. [38]

*Note:* Baseline optical properties of relevant tissues at 750 nm, derived from several literature sources.

### 2.5 Sensitivity and Modified Beer–Lambert Law

Using our abdominal model, we assessed the sensitivity of the NIRS signal to physiologic optical absorption changes in the epidermis (0–0.008 cm depth), adipose tissue (0.008 to ~2.4 cm depth), and intestinal tissue (~2.4–4 cm depth). The specific absorption coefficient changes were from 1.2 to 1.5 cm^−1^ for the epidermis, from 0.05 to 0.08 cm^−1^ for the adipose tissue, and from 0.21 to 0.24 cm^−1^ for the intestinal tissue [34, 37]. At each SDD, the FE solution of the photon diffusion equation for the fluence rate in the abdomen geometry (Section 2.1) was obtained for the baseline condition ($\phi_{0}$) and each set of perturbed conditions ($\phi_{P,i}$, where absorption in the *i*th layer is modified while absorptions in the other layers remain at baseline).

NIRS sensitivity to each tissue layer was quantified by using the semi‐infinite homogeneous tissue model to estimate the absorption change from $\phi_{P,i}$ and $\phi_{0}$ [39, 40]. This estimation, $\Delta \mu_{a,i}^{*}$, was obtained with the Modified Beer–Lambert Law [41, 42]:

(4) $$\Delta \mu_{a,i}^{*}=-\frac{\log\left(\phi_{P,i}/\phi_{0}\right)}{L},$$

where L, the differential pathlength (in cm), depends on the SDD and the baseline tissue optical properties [40, 43]:

(5) $$L\left(\mathrm{SDD}\right)\approx \frac{3\mu_{s,0}^{\prime }{\left(\mathrm{SDD}\right)}^{2}}{2\left(1+\mathrm{SDD}\times \sqrt{3\mu_{a,0}\mu_{s,0}^{\prime }}\right)}.$$

Note, $\Delta \mu_{a,i}^{*}$ is approximately the spatially averaged absorption change across the tissue probed by the NIRS measurements. We defined the NIRS sensitivity to layer *i* as the ratio of $\Delta \mu_{a,i}^{*}$ to the true absorption change ($\Delta \mu_{a,i}$), that is,

(6) $$S_{i}\left(\mathrm{SDD}\right)=\frac{\Delta \mu_{a,i}^{*}\left(\mathrm{SDD}\right)}{\Delta \mu_{a,i}}.$$

$S_{i}$ is on a scale from 0 to 1, with 1 indicating maximal sensitivity. Here, the absorption change in the intestinal layer simulates oxygenation changes in the splanchnic microvasculature supplying the intestinal tissue.

### 2.6 CNR

In addition to the sensitivity, we calculated the CNR for measurements of changes in splanchnic oxygenation as a function of SDD and measurement acquisition rate for various adipose layer thicknesses. CNR is a way to quantify the tradeoff between increased tissue depth sensitivity and decreased signal‐to‐noise with increasing SDD [39, 44, 45, 46]. The optimal SDDs for splanchnic oximetry are defined to be those with maximal CNR. For a given SDD and measurement acquisition rate, we defined the CNR using the conventional definition [45, 46]:

(7) $$\mathrm{CNR}=\frac{\phi_{P,\text{intestine}}-\phi_{0}}{\sqrt{\mathrm{var}\left(\phi_{P,\text{intestine}}\right)+\mathrm{var}\left(\phi_{0}\right)}},$$

where $\phi_{0}$ and $\phi_{P,\text{intestine}}$ are the baseline and perturbed intestinal finite‐element fluence rate solutions (see Section 2.5), and $\mathrm{var}(\phi_{0})$ and $\mathrm{var}(\phi_{P,\text{intestine}})$ are the noise‐induced variances across temporal measurements of $\phi_{0}$ and $\phi_{P,\text{intestine}}$.

Noise‐induced variances were estimated based on in vivo data collected with the same BIOPAC sensor used for the phantom test (Section 2.2). Specifically, the BIOPAC sensor was applied horizontally on the abdomen of a healthy adult at three positions near the umbilicus, and the temporal fluence rate was monitored at 1.0 cm SDD (750 nm wavelength). Note, the BIOPAC sensor only had one SDD. To mimic the effect of signal intensity decreases with increasing SDD on the noise profile, we decreased the source intensity of the BIOPAC sensor. This assumes that the noise differences between SDDs arise predominantly from their differences in signal intensity [47, 48]. At each location, monitoring was sequentially performed for five different source intensities, and at each source intensity, data was sequentially acquired at five different acquisition rates: 0.1, 0.5, 1, 5, and 10 Hz. Note, we are defining the acquisition rate as the inverse of the continuous time averaged per measurement, such that 0.1 Hz corresponds to 10‐s temporal averaging, 0.5 Hz corresponds to 2‐s temporal averaging, etc. Approximately 10 min of data were collected for each intensity, acquisition rate, and sensor location. Note, although the total measurement time was kept fixed for all acquisition rates, there were enough samples at each acquisition rate to accurately estimate the variance [49].

We then normalized the in vivo fluence rate measurements by the sensor's light coupling coefficient to tissue. The light coupling coefficient is the constant of proportionality between the measurement and finite‐element fluence rate solution; we determined it by measuring an optical phantom with known optical absorption and reduced scattering coefficients (see Section 2.2) [22]. Next, we computed the mean and standard deviation of the normalized temporal fluence rate measurements for each location, acquisition rate, and source intensity. Finally, for each acquisition rate, the means were plotted against the corresponding standard deviations, and we selected the standard deviations that correspond to the $\phi_{0}$ and $\phi_{P,\text{intestine}}$ values at each SDD via linear interpolation. We converted the two standard deviations to variances, and substituted them into Equation (7) to calculate CNR for each acquisition rate.

## 3 Results

### 3.1 Phantom Results

**FIGURE 3:**
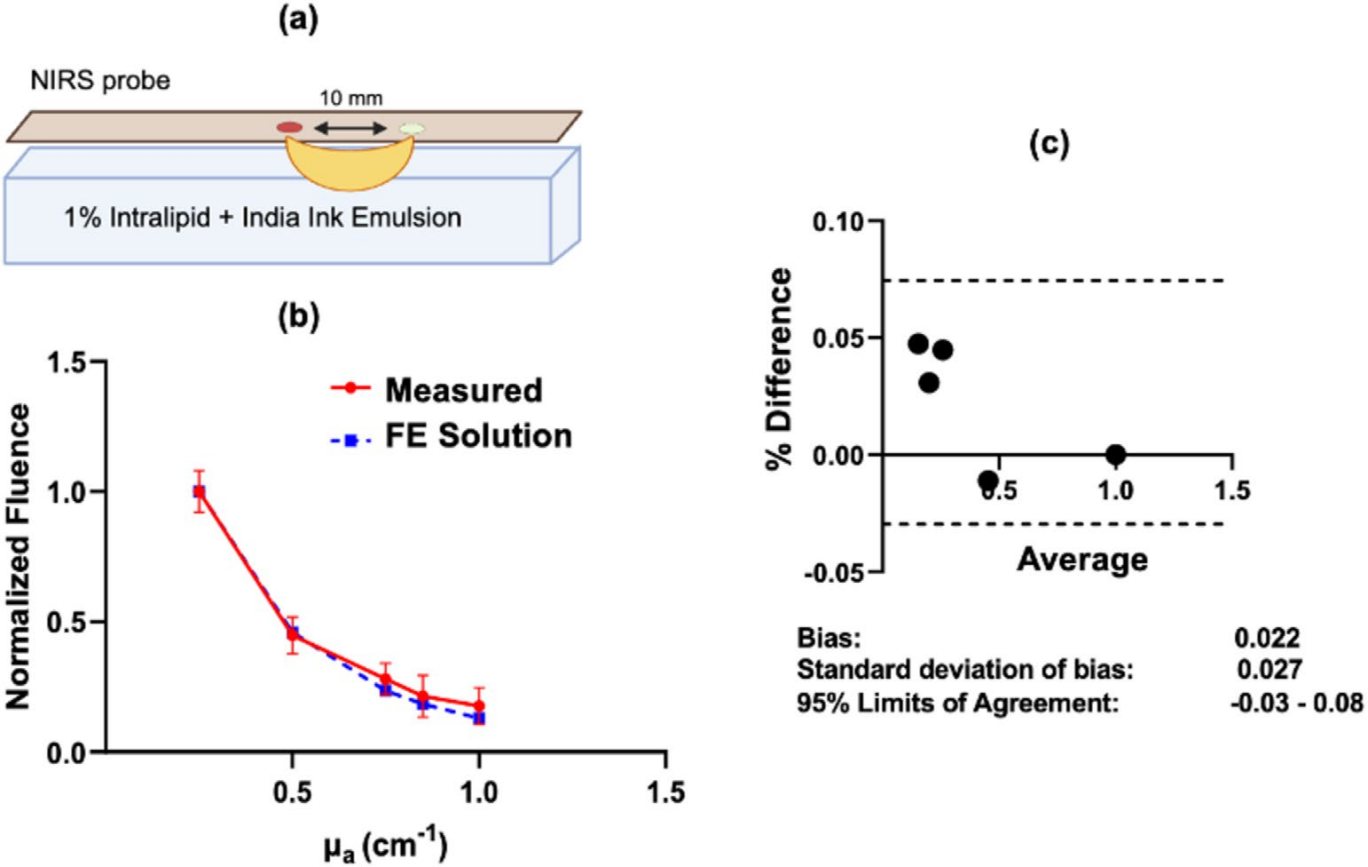
(a) Schematic of the in vitro fluence rate measurements (at 750 nm wavelength) obtained at 1 cm source–detector distance with a commercial NIRS sensor (BIOPAC Systems, red‐shaded circle is the source). (b) The in vitro fluence rate measurements (mean ± SD) and finite‐element fluence rate solutions computed for the same geometry and optical properties as the in vitro phantoms are plotted against phantom absorption coefficient (fluence rates are normalized to the values at 0.25 cm^−1^ absorption). (c) Bland–Altman plot showing the percent difference in normalized fluence values between the in vitro measurements and finite element solutions on the vertical axis, and the average of the two on the horizontal axis.

The comparison between the computed FE fluence rates and the phantom fluence rate measurements is shown in Figure 3. The data between the two models was statistically equivalent, with all computed fluence rates within one standard deviation of the fluence rate measurements for all optical absorptions (Figure 3b). The Bland–Altman plot comparing the computational and measured fluence rates is shown in Figure 3c. For all fluence rates, the difference was within the 95% limits of agreement. The observed agreement with the in vitro phantoms supports the validity of the computed finite‐element fluence rate solutions.

### 3.2 Sensitivity Analyses

**FIGURE 4:**
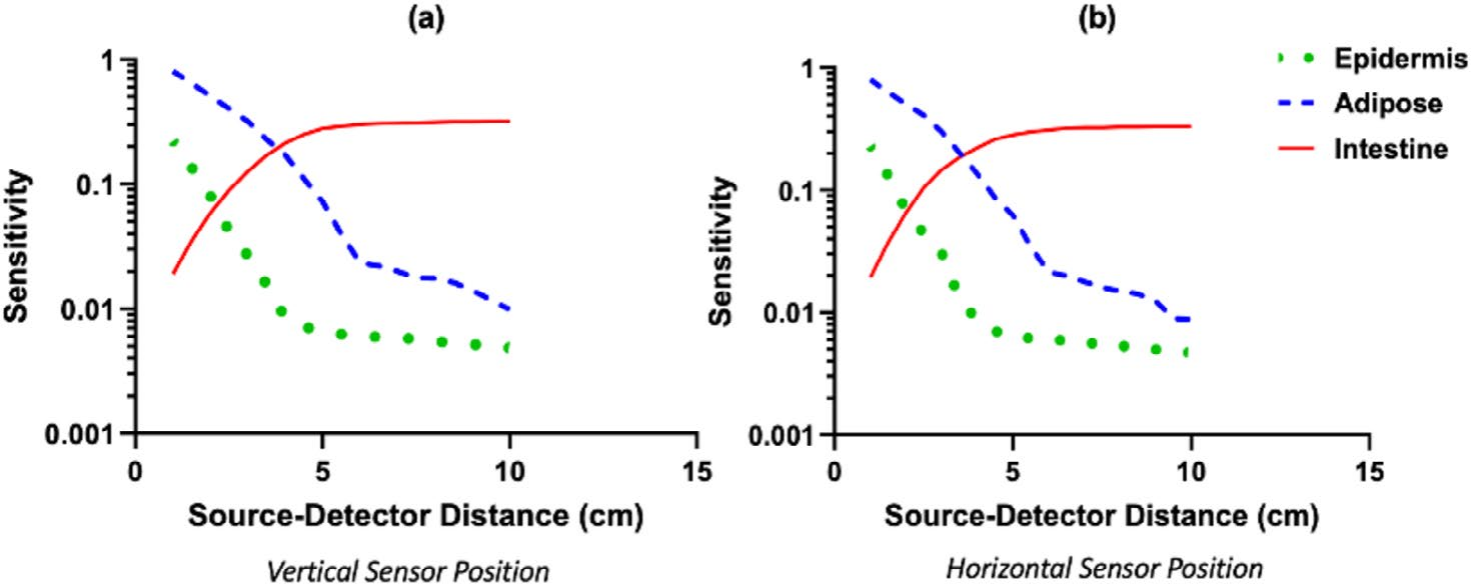
Sensitivity of abdominal near‐infrared spectroscopy to changes in epidermis, adipose, and intestine tissue absorption for the (a) vertically oriented and (b) horizontally oriented sensor positions (positions shown in Figure 2c). The sensitivities are plotted on a log scale.

Tissue sensitivities as a function of SDD for the two sensor positions simulated are shown in Figure 4 (adipose layer thickness, 2.4 cm). Between the two sensor positions tested, the differences in sensitivity were less than 5% (by direct subtraction) for all tissue types and for each SDD. For adipose tissue, sensitivity decreased monotonically with SDD, from 80% at 0.5 cm to 2% at 6 cm. Sensitivity to epidermis absorption (such as from changes in melanin content) also decreased monotonically with SDD, and was considerably lower than that of adipose tissue. For SDDs ≥ 4 cm, the sensitivity to the epidermis was nearly negligible (< 0.8%).

Conversely, sensitivity to the intestine increased with SDD, from 0.5% at 0.5 cm to 32% at 10 cm. The gain in intestinal sensitivity with increasing SDD tapers, however, for distances larger than 4.5 cm; the sensitivity at 4.5 cm was 28%.

Modest variations in adipose tissue layer thickness had a small impact on the sensitivity results (data not shown). Between the minimum and maximum thicknesses at the superior sensor position (2.0 and 2.8 cm), for example, the sensitivities to adipose tissue differed by only 5.5% at 1 cm separation, 2% at 5 cm, and 0.5% at 10 cm, while the sensitivities to intestine tissue differed by 1% at 5 cm, 2.5% at 5 cm, and 3% at 10 cm.

### 3.3 CNR and Optimal SDD

**FIGURE 5:**
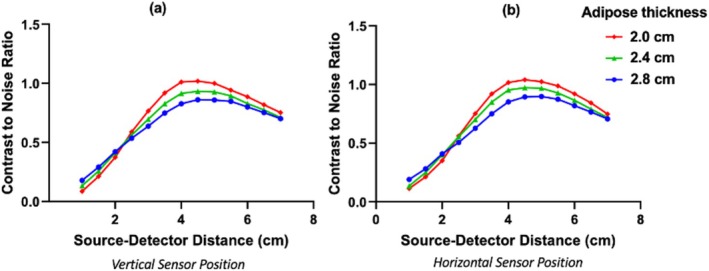
Contrast‐to‐noise ratio (CNR) of simulated abdominal near‐infrared spectroscopy fluence rate measurements of intestinal tissue absorption change at 1 Hz acquisition rate. CNR is plotted against source–detector distance for multiple adipose tissue thicknesses at the (a) vertically oriented sensor position and (b) horizontally oriented sensor position (sensor positions shown in Figure 2c).

Figure 5 shows the computed intestine CNR at 1 Hz acquisition rate plotted against SDD for each sensor position and for three adipose layer thicknesses. The CNR increased significantly with increasing SDD between 1 and 4 cm, was similar between 4 and 5 cm, and decreased with increasing SDD for distances greater than 5 cm. Thus, the CNR profiles suggest that SDDs between 4 and 5 cm are optimal for probing the infant intestine. The precise SDD modestly depends on the adipose layer thickness, for example, optimal SDD is 4.5–5 cm for 2.8 cm thickness and 4–4.5 cm for 2.0 cm thickness. The CNR itself decreases, as expected, with increasing adipose layer thickness. CNR was comparable between the two sensor positions tested.

**FIGURE 6:**
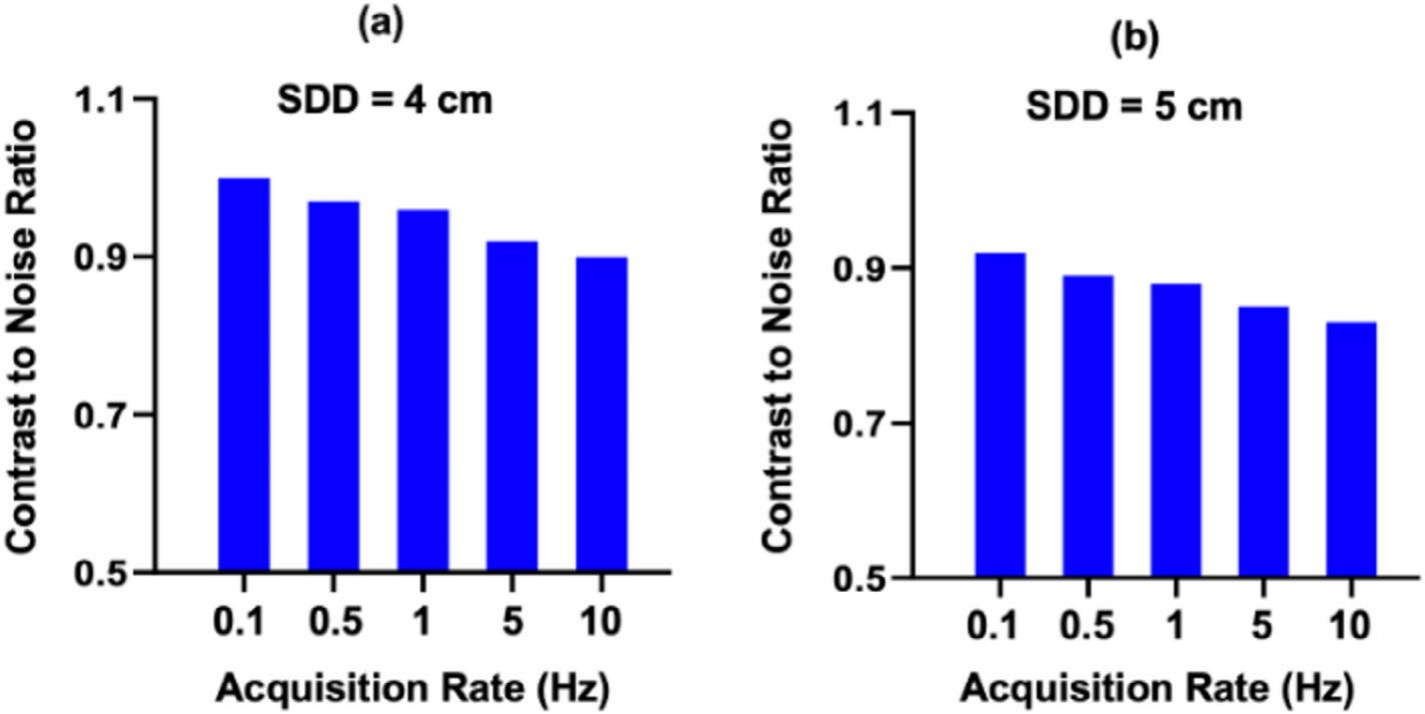
For the vertically oriented sensor position (see Figure 2c), contrast‐to‐noise ratio of simulated fluence rate measurements of intestinal tissue absorption change is plotted versus sensor acquisition rate for source–detector distances of (a) 4 cm and (b) 5 cm. Here, the acquisition rate is the inverse of the temporal averaging time (e.g., 0.1 Hz corresponds to 10‐s temporal averaging).

Figure 6 shows the effect of acquisition rate on CNR at SDD of 4 and 5 cm (for adipose layer thickness of 2.4 cm) for the vertically oriented sensor position. Note that, as expected, CNR increases with decreasing acquisition rate because of longer temporal averaging per measurement. At the lowest acquisition rate of 0.1 Hz, the CNR is 1 and 0.9 for 4 cm and 5 cm SDD, respectively.

## 4 Discussion

To facilitate sensor prototyping for infant splanchnic oximetry with abdominal NIRS, we developed a computational model to simulate abdominal NIRS signals as a function of the sensor's SDD and orientation on the abdomen. We then used this model to evaluate NIRS sensitivity and CNR for measurement of splanchnic oxygenation. The optimal SDDs for splanchnic oximetry, defined by maximal CNR, were between 4 and 5 cm. At these optimal SDDs, the NIRS signals are still sensitive to superficial adipose tissue, and the implementation of strategies to separate the intestinal signal from adipose artifacts is likely important for clinical translation. Thus, in addition to informing sensor design, the computational model can be used for testing future analysis algorithms to separate the intestinal signal from superficial tissue artifacts. We will now discuss additional insights gleaned from our simulations.

### 4.1 Sensitivity Analyses

First, the NIRS signal had minimal sensitivity to changes in epidermis absorption at longer SDDs (e.g., > 4 cm). This suggests that differences in melanin content and adipose thickness across patients will not influence the NIRS signal at these SDDs. At shorter SDDs, the impact of epidermis absorption is larger (e.g., its sensitivity at 1 cm SDD is 20%). This may have implications when shorter SDD measurements are used, for example, to separate intestine from adipose signals. Future work is needed to simulate multi‐SDD NIRS measurements for the testing of layered tissue analysis algorithms to isolate the intestinal signal.

Second, NIRS sensitivity to the intestine increased with SDD as expected. It is well known that longer SDDs are more sensitive to deeper tissues because the mean light penetration depth into the tissue is deeper [22]. In our model, intestine sensitivity exceeded that of the adipose tissue for SDD > 4 cm. Also note that intestine sensitivity increased substantially from 1 to 5 cm, but the increase tapered for longer SDDs. The shape of the intestines likely explains the tapering effect, that is, the mean light penetration depth is deeper, but the intestines are also deeper beneath the skin and thinner at large distances from the umbilicus (see Section 2.3).

Third, the sensitivities of the NIRS signals to the intestine and adipose tissues were very similar for the two sensor positions tested. This suggests that the signal sensitivity is robust to modest variability in sensor position. Nevertheless, this result is based on the assumption of a uniform change in splanchnic saturation across the intestine. For cases of localized ischemic patches, sensor position is likely important.

### 4.2 CNR

Our CNR data predicts that SDDs in the range of 4–5 cm are optimal for the infant abdomen. Nevertheless, there is a tradeoff between reduction in CNR at longer SDDs and reduction in sensitivity to superficial adipose tissue. The latter reduction, for example, helps reduce confounds from adipose changes over time and across patients. Future investigations of the costs and benefits of this tradeoff are needed, but for applications where splanchnic oxygenation is changing in isolation of superficial tissues, our results demonstrate the ability of NIRS to monitor those changes with high CNR (e.g., 1) at reasonably fast acquisition rates (e.g., 0.1 Hz). Furthermore, many clinical applications being explored for NIRS splanchnic oximetry [27, 28] do not require fast acquisition rates, and thus the use of longer acquisition times to improve CNR may be exploited.

Further, note that for SDD > 2 cm, CNR decreased with increasing adipose thicknesses, because the intestine sensitivity decreases with increasing adipose thickness. Surprisingly, however, for SDD < 2 cm, the CNR increased with increasing adipose thickness. Thus, the 2 cm SDD is a “crossing point,” where the effect of adipose thickness on CNR reverses when the SDD changes from above to below it. We note that similar crossing points have been observed in models of the effects of tissue scattering changes on the photon fluence rate in curved geometries [50, 51]. Further study is needed to better understand this effect. Measurement noise likely has an important role. At low SDD, the sensitivity of the fluence rate to absorption changes in the intestine is very small (see Figure 4). Thus, in absolute terms, the contrast (i.e., the difference in fluence rate between the perturbed and baseline intestine absorption) at low SDD will be minimally different with increasing adipose thickness. The measurement noise, however, will decrease more significantly with increasing adipose thickness, because increasing adipose thickness modestly increases the photon fluence rate, which in turn results in modestly lower relative noise. The photon fluence rate increases because the sensitivity to adipose tissue increases, and the exponential light attenuation length ($\sqrt{3\mu_{a}\left(\mu_{a}+\mu_{s}^{\prime }\right)}$ [22]) of transport through adipose tissue is smaller than that through intestine tissue. These low SDDs, however, are still clearly suboptimal for measuring the intestine (Figure 5).

### 4.3 Implications

Given the optimal SDD range of 4–5 cm suggested by our model, it is best to avoid using NIRS devices that operate exclusively with shorter SDDs for splanchnic oximetry (some pediatric NIRS systems, for example, use 2.5–3 cm SDDs [52]). Detection accuracies may further be improved by algorithms that filter artifacts from superficial adipose tissue, as was recently demonstrated in a clinical study of abdominal placental oximetry [10]. The computationally derived results here provide a means for initial testing of these algorithms with multiple sensor configurations.

Furthermore, our methods for measuring optimal sensor configuration are powerful in guiding further characterization of abdominal NIRS device design considerations, such as simulating variations in wavelength, abdominal geometry, etc. Although not considered in this study, these parameters are easily tunable using the model framework presented here, and could be easily adapted to answer a variety of significant questions surrounding abdominal NIRS optimization.

### 4.4 Limitations

Although informative, our computational results have limitations that warrant further consideration. First, a more complete in vitro validation of these results across the full 1–10 cm SDD range in layered phantoms is needed. Second, our model omits regions in the abdominal compartment where light transport is non‐diffusive, such as bowel air and fluid inside the intestines. Similarly, the epidermis in our model was very thin, which may induce errors in the approximation of diffusive light transport. Future work with methods that can account for non‐diffusive regions [53, 54] is needed to estimate the errors associated with the assumption of diffusive light transport. Third, future work is needed to study the effects from superficial tissue heterogeneities above the intestine that were not considered in our model (e.g., abdominal muscle). Notably, however, given the promising in vivo demonstrations obtained with abdominal NIRS systems that also neglect these factors [14, 15, 16], our model framework is still powerful and useful for sensor optimization.

Another limitation is that our specific reported CNR values depend on the specific NIRS sensor we used for in vivo noise estimation, that is, a BIOPAC cerebral fNIRS monitor. Other NIRS devices with different noise profiles have different CNRs. Although we expect that this device‐specific effect will only modestly influence the dependence of CNR on SDD, future work is needed to explore this further.

Finally, an important extension of our model will be the incorporation of spatially heterogeneous blood oxygen levels across the intestine. It is possible that ischemic conditions in the gut, such as those that lead to NEC, may be localized to small regions of the intestine. The sensitivity of abdominal NIRS to localized versus global oxygenation changes in the splanchnic circulation perfusing the tissue needs to be compared in future work.

## 5 Conclusion

We developed a computational method to simulate abdominal NIRS signals from infants as a function of the sensor's SDD and orientation on the abdomen. Using the model, we showed that for vertical and horizontal sensor orientations positioned near the umbilicus, NIRS measurements acquired at SDDs between 4 and 5 cm were optimal for probing splanchnic oxygenation in intestinal tissue. This computational approach for modeling abdominal NIRS signals is a useful tool for rapid sensor prototyping.

## Ethics Statement

All study procedures were approved by the Johns Hopkins University Institutional Review Board (Protocol No. IRB00316916, approved April 13, 2022).

## Consent

Patient consent was waived by the Institutional Review Board due to the use of de‐identified imaging data for this computational modeling study.

## Conflicts of Interest

The authors declare no conflicts of interest.

## Data Availability

All data supporting the findings of this study are presented within the figures of the manuscript.
